# Variable Changes of Circulating ANGPTL3 and ANGPTL4 in Different Obese Phenotypes: Relationship with Vasodilator Dysfunction

**DOI:** 10.3390/biomedicines9081037

**Published:** 2021-08-18

**Authors:** Francesca Schinzari, Giuseppina Vizioli, Umberto Campia, Manfredi Tesauro, Carmine Cardillo

**Affiliations:** 1 Department of Aging, Policlinico A. Gemelli IRCCS, 00168 Roma, Italy; francesca.schinzari@policlinicogemelli.it (F.S.); carminecardillo@unicatt.it (C.C.); 2 Department of Translational Medicine and Surgery, Catholic University, 00168 Rome, Italy; giuseppinavizioli@tiscali.it; 3 Division of Cardiovascular Medicine, Brigham and Women Hospital, Harvard Medical School, Boston, MA 02115, USA; ucampia@gmail.com; 4 Department of Systems Medicine, Tor Vergata University, 00133 Rome, Italy

**Keywords:** ANGPTL3, ANGPTL4, obesity, type 2 diabetes, endothelium, vasodilation

## Abstract

Obesity associates with premature atherosclerosis and an increased burden of cardiovascular disease, especially when accompanied by abnormalities of lipid and glucose metabolism. Angiopoietin-like (ANGPTL)3 and ANGPTL4 are metabolic regulators, whose upregulation is associated with dyslipidemia, insulin resistance and atherosclerosis. We analyzed, therefore, changes in circulating ANGPTL3 and ANGPTL4 in obese patients with different metabolic phenotypes and their relation with impaired vasodilator reactivity, an early abnormality in atherosclerosis. Compared to the lean subjects (*n* = 42), circulating ANGPTL3 was elevated (both *p* > 0.001) in the patients with metabolically unhealthy obesity (MUO; *n* = 87) and type 2 diabetes (T2D; *n* = 31), but not in those with metabolically healthy obesity (MHO; *n* = 48, *p* > 0.05). Circulating ANGPTL4, by contrast, was increased in all obese subgroups (all *p* < 0.001 vs. lean subjects). Vasodilator responses to both acetylcholine and sodium nitroprusside were reduced in the three obese subgroups vs. lean subjects (all *p* < 0.001), with greater impairment in the patients with T2D than in those with MHO and MUO (all *p* < 0.05). In the whole population, an inverse relationship (*r* = 0.27; *p* = 0.003) was observed between circulating ANGPTL4 and endothelium-dependent vasorelaxation. Circulating ANGPTL3 and ANGPTL4 undergo variable changes in obese patients with different metabolic phenotypes; changes in ANGPTL4 relate to endothelial dysfunction, making this protein a possible target for vascular prevention in these patients.

## 1. Introduction

Obesity remains a leading cause of cardiovascular morbidity and mortality [1], especially when associated with lipid abnormalities, high blood pressure or T2D [2]. Early identifications of those factors favoring the transformation of increased adiposity into a metabolic and cardiovascular disease, therefore, remains a paramount challenge for prevention.

ANGPTL3 and ANGPTL4 are proteins named according to their structural similarities with angiopoietins. ANGTPL3 is produced and released into systemic circulation almost exclusively by the liver [3], whereas ANGPTL4 is expressed ubiquitously, with a high degree of expression occurring in the adipose tissue [4]. Among a variety of biological functions, ANGPLT3 and ANGPLT4 exert a coordinate action to regulate lipid metabolism, predominantly by the inhibition of LPL in response to changes in nutritional state [5,6]. Not surprisingly, transgenic mice with overexpression of *Angptl3* or *Angptl4* have increased plasma triglyceride levels [5,6]; knockout animals, by contrast, have reduced levels of triglyceride-rich lipoproteins and increased LPL activity [7,8]. In keeping with these experimental observations, population-based studies have shown that loss-of-function variants of either *Angptl3* or *Angptl4* are associated with lower plasma triglycerides and a decreased risk of cardiovascular disease [9,10]. The influence of these proteins on glucose metabolism is less well-defined. Studies in animal models with target deletion of *Angptl3* have indicated that a reduction in ANGPTL3 improves insulin sensitivity [11,12], but this effect seems secondary to improved lipid metabolism, rather than to the direct action of ANGPTL3. With regard to ANGPTL4, studies in knockout mice or in humans with loss-of-function alleles of *Angptl4* have shown no changes in plasma glucose or insulin levels [8,13]. A recent study, however, has demonstrated an improved glucose tolerance accompanied by an increased fat mass in *Angptl4* knockout mice fed a diet high in saturated fatty acids, suggesting that an increase in LPL activity in the adipose tissue might prevent insulin resistance in these animals [14].

One mechanism by which abnormalities in lipid and glucose metabolism may initiate the process leading to vascular damage and cardiovascular events is the impairment of endothelial function [15]. Thus, endothelial dysfunction, defined as decreased vasodilator reactivity to stimuli eliciting the release of vasodilator substances by vascular endothelium, NO in predominance, is undisputedly recognized as the earliest inciting event in atherosclerosis [16]. A more accurate characterization of the factors involved in this abnormality, therefore, might translate into better opportunities for treatment. 

The present study tested the hypothesis that changes in the circulating concentrations of ANGPTL3 and ANGPTL4 might occur in obese patients according to their disparate metabolic phenotypes, ranging from MHO, through MUO, to T2D. Another purpose of the study was to assess the relationship of circulating levels of ANGPTL3 and ANGPTL4 with the abnormalities in vasodilator reactivity observed in the various obese subgroups.

## 2. Methods

### 2.1. Analytical Procedures 

Participants were asked to fast for at least 8 hours before the study. Then, while the participant was supine, a deep antecubital vein was cannulated with a 20-gauge catheter (Abbott Laboratories, Abbott Park, IL, USA); blood was collected in citrated tubes and immediately spun in a refrigerated centrifuge; plasma samples were aliquoted to avoid multiple freeze/thaw cycles that may reduce the stability of the analytes and then immediately stored at −80 °C; aliquots used for this study where thawed immediately before the assay and carefully inspected for their conformity, to rule out gross hemolysis or abnormalities in the preservation. Measurements of insulin, ANGPTL3 and ANGPTL4 were performed on stored plasma samples using Luminex assays (R&D Systems, Minneapolis, MN, USA). Insulin sensitivity was determined by use of the HOMA index of insulin resistance [17].

### 2.2. Vascular Reactivity Studies

Each study consisted of infusions of drugs into the brachial artery and measurement of forearm blood flow by means of strain-gauge venous occlusion plethysmography. All drugs used in this study were prepared by the hospital research pharmacy following specific procedures to ensure accurate bioavailability and sterility of the solutions. While participants were supine, a 20-gauge Teflon catheter (Arrow Inc., Limeric, PA, USA) was inserted into the brachial artery of the non-dominant arm (left in most cases) for drug infusion. The extended arm was positioned slightly above the level of the right atrium and a mercury-filled strain gauge was placed around the widest part of the forearm. The strain gauge was connected to a plethysmograph (model EC-6, Hokanson Inc., Bellevue, WA, USA) calibrated to measure the percent change in volume and connected to a personal computer through an analog-to-digital converter. For each measurement, a cuff placed around the upper arm was inflated to 40 mm Hg with a rapid cuff inflator (model E-10, Hokanson) to occlude venous outflow from the extremity. A wrist cuff was inflated to suprasystolic pressures 1 minute before each measurement to exclude the hand circulation. Flow measurements were recorded for approximately 7 s every 15 s; 7 readings were obtained for each mean value. Blood pressure was recorded with the use of a standard mercury manometer. Throughout all studies, volumes infused were matched by administration of variable amounts of saline.

Vasodilator reactivity was tested as previously reported in detail [18]. Briefly, after the forearm was instrumented, saline was infused intra-arterially for 15 min, basal flow was measured and dose–response curves to acetylcholine chloride (ACh, Sigma-Aldrich, St. Louis, MO, USA), which is known to induce vasodilation through, at least in part, endothelial release of NO, and to the exogenous NO donor sodium nitroprusside (SNP, Malesci, Florence, Italy) were obtained. The sequence of ACh and SNP infusion was randomized to avoid bias related to the order of these procedures. 

### 2.3. Statistical Analyses

Group comparisons were performed by 1-way and 2-way ANOVA, as appropriate; the Holm–Sidak test was used for post hoc comparisons when needed. Univariate and multivariate analyses of association on the combination of all groups (patients and controls) were tested by use of standard linear regression analysis and multiple backward stepwise linear regression on variables with significant results at the univariate regression. When data were not normally distributed, non-parametric tests were applied or data were analyzed after rank transformation. All calculated probability values are 2-tailed, and a *p* value < 0.05 was considered statistically significant. All group data are reported as mean ± SEM. 

### 2.4. Participants

Healthy, lean subjects (BMI < 25 kg/m^2^, normal waist circumference) and patients with central obesity (waist circumference ≥ 102 cm for males or ≥ 88 cm for females) were included in the study. Obese patients were classified as having MHO in the absence of any of the metabolic abnormalities defining the metabolic syndrome according to the ATP III criteria [19] or as having MUO in the presence of at least one of those abnormalities; those obese patients who fulfilled the criteria set by the American Diabetes Association for the diagnosis of diabetes [20] were included in another, separate group. Exclusion criteria were history or current evidence of cardiovascular disease (coronary artery disease, cerebrovascular or peripheral occlusive arterial disease, coagulopathy, vasculitis) or any other systemic condition. In obese and diabetic patients taking antihypertensive and/or lipid-lowering drugs, treatment was discontinued for at least one week prior to the study. During this time, blood pressure and plasma glucose levels were repeatedly measured and, when needed, treatment was resumed, and the patient excluded from the study. None of the participants were smokers and all of them were asked to refrain from drinking alcohol and beverages containing caffeine for at least 24 h before the study. None of the participants was taking vitamin supplements or engaged in programs of regular physical activity. The study protocols were approved by the local Institutional Review Board and all participants gave written informed consent before their involvement in the study. 

## 3. Results

The baseline anthropometric, hemodynamic and biochemical characteristics of the participants are reported in Table 1. All the obese patients had a measure of waist circumference above the ATP III criteria for central obesity [19], hence indicating the presence of visceral adiposity.

### 3.1. Group Differences in ANGPTL3 and ANGPTL4

**Figure 1:**
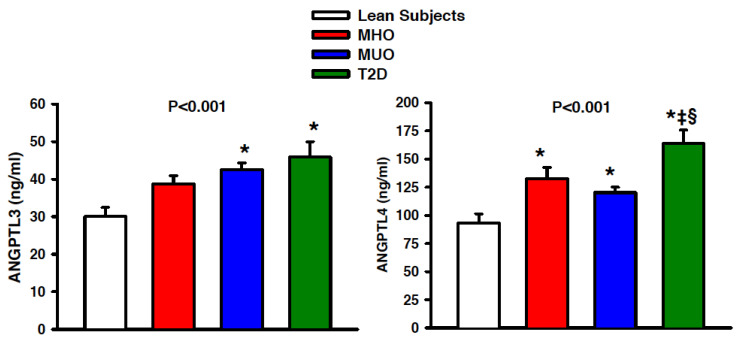
Graphs showing plasma concentrations of angiopoietin-like (ANGPTL) 3 (left panel) and ANGPTL4 (right panel) in lean subjects (open bars), patients with metabolically healthy obesity (MHO, red bars), patients with metabolically unhealthy obesity (MUO, blue bars) and obese patients with type 2 diabetes (T2D, green bars). The *p* values refer to the comparisons among the 4 groups by 1-way analysis of variance. All values are means ± SEM. * *p* < 0.05 vs. lean subjects, ^‡^ *p* > 0.05 vs. MHO and ^§^ *p* > 0.05 vs. MUO at the post hoc Holm–Sidak test for multiple comparisons.

In comparison to the lean subjects, the circulating levels of ANGPTL3 were similarly elevated in the patients with MUO and T2D, but not in those with MHO; the difference between the patients with MUO or T2D and those with MHO, however, was not statistically significant (Figure 1, left panel). The circulating levels of ANGPTL4 were increased in all the obese subgroups (MHO, MUO and T2D) compared to the lean subjects; of note, plasma ANGTPL4 was higher in the patients with T2D than in the other obese subgroups (MHO and MUO), whereas the difference between those with MHO and MUO was not statistically significant (Figure 1, right panel). 

### 3.2. Group Differences in Vasodilator Reactivity 

An assessment of the vascular response to ACh was obtained in 125 study participants (20 lean subjects, 24 patients with MHO, 55 patients with MUO and 26 patients with T2D); an assessment of the vascular response to SNP was obtained in 99 study participants (19 lean subjects, 16 obese patients with MHO, 38 patients with MUO and 26 patients with T2D). 

**Figure 2:**
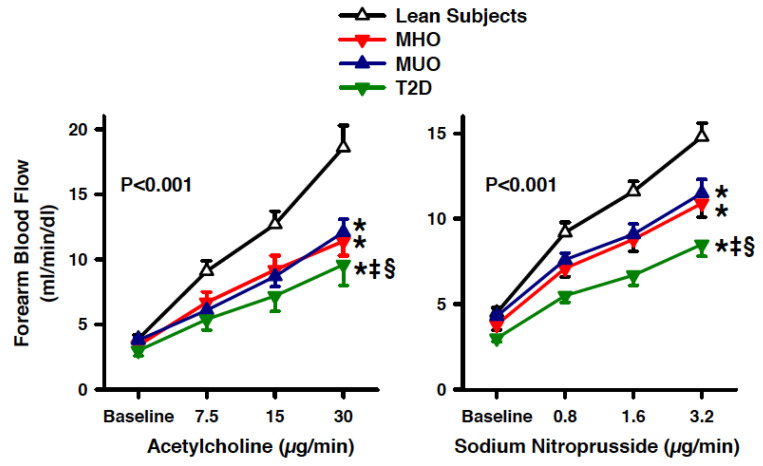
Graphs showing the forearm blood flow responses to acetylcholine (left panel) and sodium nitroprusside (right panel) in lean subjects (open triangles), patients with metabolically healthy obesity (MHO, red triangles), patients with metabolically unhealthy obesity (MUO, blue triangles) and obese patients with type 2 diabetes (T2D, green triangles). The *p* values refer to the comparisons among the 4 groups by 2-way analysis of variance. All values are means ± SEM. * *p* < 0.05 vs. lean subjects, ^‡^ *p* > 0.05 vs. MHO and ^§^ *p* > 0.05 vs. MUO at the post hoc Holm–Sidak test for multiple comparisons.

An infusion of graded doses of ACh or SNP resulted in a progressive vasodilator response in all the groups (all *p* < 0.001 vs. baseline). The vasodilator reactivity to ACh, however, was significantly reduced in the three obese subgroups (MHO, MUO and T2D) compared to lean subjects; of note, the vasodilation induced by ACh was lower in the patients with T2D, even in comparison to the other obese subgroups (MHO and MUO), whereas no difference was observed between the patients with MHO and MUO (Figure 2, left panel). Similar to the ACh results, the vasodilator response to SNP was reduced in all of the three obese subgroups (MHO, MUO and T2D) compared to the lean participants; also, the vasodilation induced by SNP was lower in the patients with T2D, even in comparison to the other obese subgroups (MHO and MUO), whereas no difference was observed between the patients with MHO and MUO (Figure 2, right panel).

### 3.3. Correlations of Circulating ANGPT3 and ANGPT4

**Figure 3:**
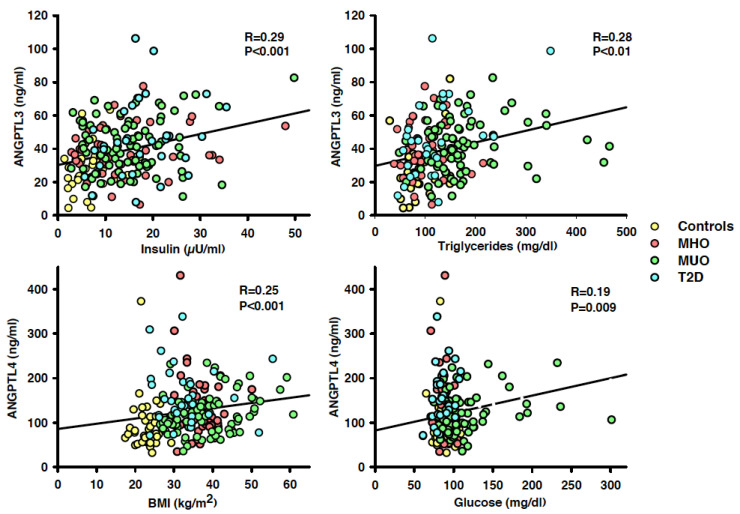
Graphs showing the linear relationships in the whole population of circulating ANGPTL3 with plasma insulin (left top panel) and triglycerides (right top panel), and of ANGPTL4 with body mass index (BMI; left bottom panel) and plasma glucose (right bottom panel). At the multivariate regression analysis, insulin and triglycerides remained the only variables bearing a significant linear association with circulating ANGPTL3, whereas body mass index and plasma glucose remained the only variables bearing a significant linear association with circulating ANGPTL4. The *r* values indicate the regression coefficients, and the *p* values indicate the level of significance at the linear regression analysis.

In the whole population, circulating ANGPTL3 had a linear, direct association with BMI (*r* = 0.26, *p* < 0.001), MAP (*r* = 0.21, *p* = 0.004), plasma concentrations of insulin (*r* = 0.29, *p* < 0.001; Figure 3, left top panel), glucose (*r* = 0.15; *p* = 0.03), LDL cholesterol (*r* = 0.15, *p* = 0.04) and triglycerides (*r* = 0.28, *p* = 0.001; Figure 3, right top panel); no significant association of circulating ANGPTL3, by contrast, was observed with age and HDL cholesterol (both *p* > 0.05). In a multivariate regression model including the variables with a significant linear relation with ANGPTL3 at the univariate regression analysis, insulin (*p* < 0.001) and triglycerides (*p* = 0.005) remained associated in linear combination with circulating ANGPTL3 (*r* = 0.38, *p <* 0.001).

**Figure 4:**
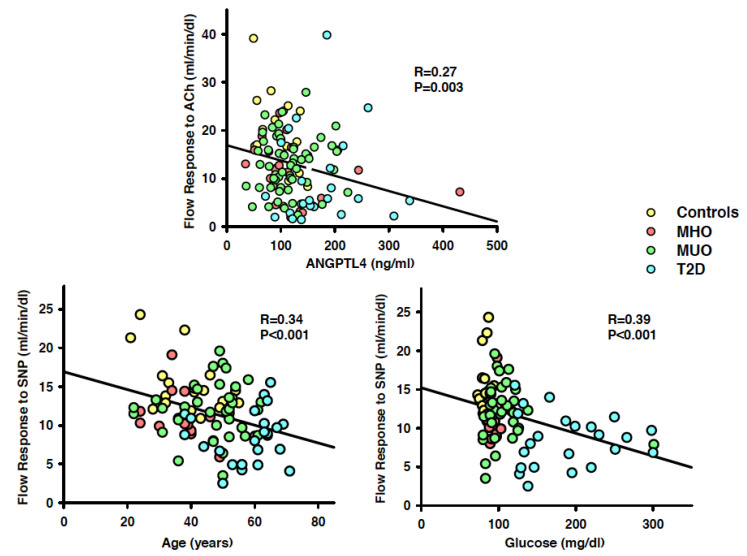
Graphs showing the linear relationships in the whole population of the forearm blood flow responses to acetylcholine (ACh) with circulating ANGPTL4 (top panel), and of the forearm blood flow responses to sodium nitroprusside (SNP) with age (left bottom panel) and plasma glucose (right bottom panel). At the multivariate regression analysis, ANGPTL4 remained the only variable bearing a significant linear association with the vasodilator effect of ACh, whereas age and plasma glucose were the only variables bearing a significant linear association with the vasodilator effect of SNP. The *r* values indicate the regression coefficients, and the *p* values indicate the level of significance at the linear regression analysis.

Circulating ANGPTL4 had a linear, direct relationship with BMI (*r* = 0.26, *p* < 0.001; Figure 3, left bottom panel), plasma insulin (*r* = 0.18, *p* = 0.01) and glucose (*r* = 0.28, *p* < 0.001; Figure 4, right bottom panel); no significant association, by contrast, was observed between ANGPTL4 and all the other variables (all *p* > 0.05). At the multivariate regression analysis, BMI (*p* = 0.02) and plasma glucose (*p <* 0.001) remained associated in linear combination with circulating ANGPTL4 (*r* = 0.41, *p* < 0.001). 

No linear relationship was observed between the plasma levels of ANGPTL3 and ANGPTL4 (*r* = 0.06, *p* = 0.43).

### 3.4. Correlations of Vascular Responses to ACh and SNP

In the whole population, the vasodilator response to ACh showed an inverse, linear relationship with plasma glucose (*r* = 0.25, *p* = 0.007) and ANGPTL4 (*r* = 0.27, *p* = 0.003; Figure 4, top panel), but had no significant relation with age, MAP, plasma insulin, glucose, triglycerides, LDL cholesterol, HDL cholesterol and ANGPTL3 (all *p* >0.05). In a multivariate regression model including the variables bearing a significant linear relationship with the vasodilator effect of ACh at the univariate regression analysis, only ANGPTL4 remained associated in linear combination with endothelium-dependent vasodilation (*r* = 0.28, *p* = 0.002).

The vasodilator response to SNP showed an inverse, linear relationship with age (*r* = 0.34, *p* < 0.001; Figure 4, left bottom panel), BMI (*r* = 0.23, *p* = 0.02), plasma insulin (*r* = 0.25; *p* = 0.02), glucose (*r* = 0.39, *p* < 0.001; Figure 4, right bottom panel) and ANGPTL4 (*r* = 0.29, *p* = 0.004), but had no linear association with all the other variables (all *p* > 0.05). At the multivariate regression analysis, age (*p* = 0.02) and plasma glucose (*p* = 0.005) remained associated in linear combination with endothelium-independent vasodilation (*r* = 0.46, *p* < 0.001).

## 4. Discussion

An important finding of the current study is that the circulating concentrations of ANGPTL3 and ANGPTL4 undergo variable changes in obese patients, according to their different metabolic phenotypes. Thus, compared to the lean subjects, circulating ANGPTL3 was significantly elevated only in the obese patients with metabolic abnormalities, such as those with MUO or T2D, but not in those with MHO. An increase in circulating ANGPL4, by contrast, was observed in all the obese phenotypes, irrespective of the absence or presence of metabolic abnormalities, even though the rise in circulating ANGPTL4 was more pronounced in the patients with T2D than in those with MHO and MUO. Taken together, these findings suggest that different mechanisms are involved in the dysregulation of ANGPTL3 and ANGPTL4 in patients with increased adiposity or, alternatively, that diverse metabolic abnormalities stem from the obesity-associated changes in circulating ANGPTL3 and ANGPTL4.

Previous studies measuring plasma concentrations of ANGPTL3 in insulin-resistant states, such as obesity and type 2 diabetes, have yielded conflicting results. The majority of investigations have reported increased circulating ANGPTL3 in patients with obesity or T2D [21,22,23], whereas other studies have found that the plasma levels of ANGPTL3 are not higher in obese individuals than in controls [24,25]. The differences in the metabolic characteristics of the patients included in the various studies might have contributed to their discrepant outcomes, as suggested by our observation that, beyond obesity per se, the coexistence of metabolic abnormalities is associated with a substantial rise in circulating ANGTPL3. As to the mechanisms leading to increased circulating ANGPTL3, our and previous results hint toward a possible involvement of hyperinsulinemia. Thus, even though hyperinsulinemia during euglycemic clamp has been shown to acutely suppress plasma ANGPTL3 concentrations in obese non-diabetic patients, prolonged hyperinsulinemia increases the ANGPTL8 RNA and protein concentrations in cell cultures of hepatocytes and adipocytes [26]; because the secretion of ANGPTL3 and ANGPTL8 is mechanistically interdependent [27], it is possible that the stimulation of ANGPTL8 production by chronic hyperinsulinemia might result in the concurrent increase in circulating ANGPTL3 [3]. With regard to the direct relationship observed in our study between increased plasma levels of ANGPTL3 and triglycerides, its most likely explanation might deal with the primary biological action of ANGPTL3 to inhibit LPL, hence affecting the clearance of triglyceride-rich lipoproteins in the capillary bed of various tissues, such as the adipose tissue and the skeletal muscle [3]. Previous studies, however, have not provided univocal findings in this regard. In fact, whereas markedly lower plasma triglycerides have been observed in subjects with a loss of function mutation of *Angptl3* [13] and in genome-wide association studies linking plasma triglycerides to specific loci near *Angptl3* [28], other studies have reported an absent or even an inverse association between circulating triglycerides and ANGPTL3 [29,30]. The precise reasons for these discrepancies remain unclear, even though the clinical characteristics of the participants in different studies might have certainly played a role. 

In our study, in contrast to ANGPTL3, the changes in circulating ANGTL4 were not related to abnormalities in lipid metabolism but resulted as associated with those in glucose homeostasis. Thus, the circulating levels of ANGPTL4 were significantly higher in the group of patients with T2D than in any other group, including those with MHO and MUO, with a pattern of changes reproduced exactly by the HOMA index of insulin resistance. Taken together, these observations give rise to the hypothesis that ANGPTL4 might have a mechanistic function in the development of obesity-related insulin resistance and hyperglycemia. This assumption lends support from experimental studies in the loss-of-function models of *Angptl4*, displaying improved glucose tolerance compared to their wild-type counterparts [14,31], and in transgenic mice with *Angptl4* overexpression, exhibiting, by contrast, a reduced glucose utilization compared to controls [32]. Additionally, human studies have demonstrated that individuals with a missense variant (E40K) of *Angptl4* have higher insulin sensitivity following a glucose-tolerance test, with reduced probability to develop T2D [33]. In addition, the upregulation of circulating ANGPTL4 in obese patients with T2D has been reported by a large population study, demonstrating a positive correlation between plasma ANGPTL4 concentrations and fasting plasma glucose [29], and by another investigation, showing that the plasma levels of ANGPTL4 are almost two-fold higher in patients with T2D than in nondiabetic controls [34].

### 4.1. Novelty of the Study

The main novel finding of our study is the relationship observed between the circulating concentrations of ANGPTL4 and the obesity-associated endothelial dysfunction. Compared to the lean subjects, impaired vasodilator responses to both ACh and SNP were observed in all our obese subgroups; the defect in vasodilator reactivity, however, was more pronounced in the patients with T2D than in the other two subgroups (MHO and MUO). Our observation that obesity and T2D result in reduced vasodilator responsiveness to both Ach and SNP is in line with previous data, suggesting that these insulin resistant states, especially T2D, derail not only endothelial function, but also smooth muscle responsiveness to NO [35]. Interestingly, in the present study, among several variables bearing a linear, inverse association with the vasodilator reactivity to ACh at univariate regression analysis, including BMI, plasma insulin, glucose and ANGPTL4 (but not ANGPTL3), only ANGPTL4 remained significantly associated with endothelium-dependent vasorelaxation at the multivariate regression. This observation supports the view that an elevation of ANGPTL4 might play a deterministic role in the endothelial dysfunction of these patients. Previous studies have demonstrated that the loss-of-function mutations in *ANGPTL4* are associated with protection against coronary artery disease, indirectly hinting at a role of ANGPTL4 in atherosclerosis [36]. In this regard, the results of the current study may provide a potential link between ANGPTL4 and atherosclerosis, given that a defect in the endothelium-dependent vasodilator function is considered the earliest stage in the atherogenic process [15,16]. A putative mechanism by which ANGPTL4 might evoke endothelial dysfunction involves hyperglycemia, as suggested by an investigation showing that the genetic ablation of ANGPTL4 in mouse adipose tissue not only improves glucose tolerance, but also reduces atherosclerosis [37], hence underscoring the relevant influence of ANGPTL4 on both glucose metabolism and vascular disease. This notion has been recently strengthened by a study that examined the effects of the genetic loss-of-function variants of *Angptl3* and *Angptl4*, showing that genetic mimicry of ANGPTL4 inhibition is associated with an improved insulin-glucose metabolism and a lower risk of T2D and coronary artery disease [9].

### 4.2. Limitations of the Study

It must be acknowledged that because our study was performed in the intact human circulation, it is inherently difficult to ascertain whether factors other than hyperglycemia play a role in the pathophysiology of endothelial dysfunction in the context of elevated ANGPTL4 and, more in general, to identify the precise molecular mechanisms involved. Another limitation of the present study relates to its cross-sectional design, which allows us to detect associations, but not to gain deterministic insights.

## 5. Conclusions and Perspectives

Irrespective of these considerations, our data clearly suggest that changes in circulating ANGPTL3 or ANGPTL4 herald some obesity-related abnormalities, such as dyslipidemia or the dysregulation of glucose and vascular homeostasis. Further studies, however, are needed to ascertain whether ANGPTL3 and ANGPTL4 play a mechanistic function in linking increased adiposity to its metabolic and vascular sequelae. Considering the efficacy shown in recent studies by therapies targeting these proteins, including antisense oligonucleotides and monoclonal antibodies [38,39], the demonstration of an involvement of ANGPTL3 and ANGPTL4 in the pathophysiology of the complications of obesity could translate into more effective, personalized strategies for prevention. 

## Figures and Tables

**Table 1:** Clinical Characteristics of the Study Population.

	Lean Subjects (*n* = 42)	MHO (*n* = 48)	MUO (*n* = 86)	T2D (*n* = 31)	*p* Value
Sex, m/f	19/23	18/30	53/33	15/16	-
Age, yr	42 ± 2	38 ± 1	46 ± 1 ^‡^	56 ± 2 ^§^	<0.001
BMI, kg/m^2^	23 ± 1	37 ± 1 *^†^	38 ± 1 *^†^	33 ± 1 *	<0.001
Waist, cm	81 ± 2	118 ± 2 *	118 ± 2 *	111 ± 3 *	<0.001
MAP, mmHg	86 ± 1	94 ± 1 *^†^	107 ± 1 ^§^	84 ± 2	<0.001
Glucose, mg/dL	86 ± 1	90 ± 1	112 ± 4 *^‡^	171 ± 11 ^§^	<0.001
Total Cholesterol, mg/dL	173 ± 4	182 ± 5	204 ± 6 ^§^	167 ± 8	<0.001
LDL Cholesterol, mg/dL	109 ± 4	113 ± 4	133 ± 6 ^§^	103 ± 7	0.002
HDL Cholesterol, mg/dL	50 ± 2	50 ± 2	42 ± 2 *^‡^	45 ± 2	0.008
Triglycerides, mg/dL	92 ± 8	97 ± 5	182 ± 12 ^§^	116 ± 11	<0.001
Insulin, μU/mL	7 ± 1	14 ± 2 *	15 ± 1 *	18 ± 3 *	<0.001
HOMA	1.5 ± 0.2	3.2 ± 0.3 *	4.6 ± 0.5 *	7.3 ± 1.5 ^§^	<0.001

Data are Expressed as Mean ± SEM. Comparisons were performed using a one-way analysis of variance. MHO indicates metabolically healthy obesity; MUO indicates metabolic unhealthy obesity; T2D indicates type 2 diabetes; BMI, body mass index; MAP, mean arterial pressure; LDL, low-density lipoprotein; HDL, high-density lipoprotein; HOMA, HOMA index of insulin resistance. * *p* < 0.05 vs. lean subjects, ^‡^ *p* < 0.05 vs. MHO, ^†^ *p* < 0.05 vs. T2D, ^§^ *p* < 0.05 vs. all other groups at a one-way analysis of variance with a Holm–Sidak post hoc test for multiple comparisons; - means that statistical analysis on group differences in gender was not performed.

## Data Availability

The data that support the findings of this study are available from the corresponding author upon reasonable request.

## Abbreviations

Ach	Acetylcholine
ANGPTL	Angiopoietin-like
ATP	Adult Treatment Panel
BMI	Body Mass Index
HDL	High-Density Lipoprotein
HOMA	Homeostasis Model Assessment
LDL	Low-Density Lipoprotein
LPL	Lipoprotein Lipase
MAP	Mean Arterial Pressure
MHO	Metabolically Unhealthy Obesity
MUO	Metabolically Healthy Obesity
NO	Nitric Oxide
SNP	Sodium Nitroprusside
T2D	Type 2 Diabetes
